# Calendar

**DOI:** 10.1155/S0962935196000634

**Published:** 1996-12


					Calendar
Calendar
1996
1-5 December 1996
Inaugural Conference of the Federation of
Immunological Societies of Asia--Oceania
(FIMSA) in conjunction with the 24th
Annual Scientific Meeting of the
Australasian Society for Immunology (ASI),
Adelaide, Australia (contact: FIMSA 96
Conference Secretariat, PO Box 153,
Nairne, SA, Australia, 5252, Tel/Fax: 61 8
388 6164 or E-mail:
Ident@microb.adelaide,edu.au)
4-6 December 1996
New Trends in Atherosclerosis Research,
Paris, France.
Contact: L. Drye, Institut Pasteur
Eurconferences, 28 rue du Docteur
Roux 75724, Paris Cedex 15, France.
Fax: (+33) (1) 40 61 34 05.
5-8 December 1996
16th European Workshop on
Inflammation: Inflammation and Skin,
Leipzig, Germany.
Contact: Prof. Dr M. Kletzmann,
Institut ftir Pharmakologie, Pharmazie und
Toxikologie, Veteriniirmedizinische
Fakuldit, Universitiit Leipzig,
Zwickauer Str. 55,
D-04103 Leipzig, Germany.
Fax: (+49) 341 9738149.
or
Prof. Dr G. Wozel
Klinik ftir Hautkrankhelten,
Medizinische Akademie Carl Gustav Carus,
Fetscherstr. 74, D-O1307 Dresden,
Germany.
Fax: (+49) 351 4584338.
1997
24-25 February 1997
Third Annual Anti-inflammatory Drug
Discovery Summit, Princeton, NJ, USA.
Contact: Mark Alexay, Strategic Research
Institute.
Tel: 800-599-4950 ext 251.
17-19 March 1997
International Conference on
Inflammopharmacology with Vth
Symposium on 'Side Effects of Anti
inflammatory Drugs' San Francisco, CA,
USA.
Contact: Dr Sherwood Reichard, Meeting
Manager, MAPS, Suite 9, 1021 15th Street,
Augusta, GA 30901, USA.
Fax: 706-722-7515.
E-mail: maps@csra.net
or
Professor K.D. Rainsford, ICI-SGADS
Meeting Division of Biomedical Sciences,
Sheffield Hallam University, Pond Street,
Sheffield S1 lWB, UK.
Fax: (+44) 114 532 2020
E-mail: k.d.rainsford@she.ac.uk
21-25 May 1997
Fourth International Symposium on the
Immunotherapy of the Rheumatic
Diseases, Cyprus.
Contact: Symposium Secretary,
Rheumatology Unit, 4th Floor Hunt's
House, Guy's Hospital, London SE1 9RT,
UK.
Tel: 0171-955-4394
Fax: 0171-955-2472.
7-9 June 1997
Third OARS International Congress
'Osteoarthritis in Focus: Etiopathogenesis,
Assessment & Treatment', Singapore.
Contact: Evie Altman-Orbach, P.O. Box
30096, Bethesda, MD 20824, USA.
Tel: 301-718-1836
Fax: 301-913-2805
E-mail: oarsorg@aol.com
16-20 November 1997
The 3rd World Congress on Inflammation,
Tokyo, Japan.
Contact: The Secretariat,
c/o PMSI Japan Ltd, Royal Bldg 12-8
Nibancho, Chiyoda-ku, Tokyo 102, Japan.
Fax: (+81) 3 5275 6994.
19-22 November 1997
Xth EULAR Symposium: New Anti-
inflammatory and Immuno-modulating
Agents: Clinical and Experimental
aspects, Benefits and Risks, Vienna,
Austria.
Contact: EULAR Secretariat,
Witikonerstrasse 15, CH-8032, Zurich,
Switzerland.
Tel: (+41) 383 96 90
Fax: (+41) 383 98 10.
3-5 December 1997
BSI Fifth Annual Congress, Brighton.
BSI, Triangle House, Broomhill Road,
London SWl8 4HX.
Tel: 0181 877 9920
Fax: 0181 877 9308
E-mail: bsi@immunology.org
1998
20-25 April 1998
5th International Conference on Systemic
Lupus Erythematosus, Cancun, Mexico.
Further information from Dr Donato
Alarc6n Segovia, Director General,
Instituto Nacional de la Nutricion,
Salvador Zubiran, Vasco de Quiroga 15,
Delegaci6n Tlalpan, C.P. 14000
Mexico D.E
Tel: (525) 655 5954/513 0082
Fax: (525) 573 2096
E-mail: jalcocee@condor.dgsca.unam.mx
21-26 June 1998
XIIth Pan-American Congress of
Rheumatology, Montreal.
Contact: PANLAR, c/o Sorelcomm (1985)
Inc., 4446 St Laurent Boulevard, Suite 704,
Montreal, Quebec, Canada H2W 1Z5.
Tel: +514 499 8920
Fax: +514 499 8921.
23-26June 1998
XIth EULAR Symposium, Atypical
Arthritides: A Multi-disciplinary Approach,
Geneva, Switzerland.
Contact: EULAR Secretariat,
Witikonerstrasse 15, CH-8032, Zurich,
Switzerland.
Tel: (+41) 383 96 90
Fax: (+41) 383 98 10
16-18 December 1998
BSI Sixth Annual Congress, Harrogate.
BSI, Triangle House, Broomhill Road,
London SWl8 4HX.
Tel: 0181 877 9920
Fax: 0181 877 9308
E-mail: bsi@immunology.org
(C) 1996 Rapid Science Publishers Mediators of Inflammation Vol 5 1996 453

				

